# Neuropsychological effects of chronic low-dose exposure to polychlorinated biphenyls (PCBs): A cross-sectional study

**DOI:** 10.1186/1476-069X-4-22

**Published:** 2005-10-19

**Authors:** Martin Peper, Martin Klett, Rudolf Morgenstern

**Affiliations:** 1 Charité Universitätsmedizin Berlin, Institute of Pharmacology and Toxicology (CCM), Dorotheenstr. 94, 10117 Berlin, Germany; 2 Public Health Unit Rhein-Neckar-Kreis, State of Baden-Württemberg, Kurfürstenanlage 38-40, 69115 Heidelberg, Germany; 3 Present address: University of Freiburg, Department of Psychology, Belfortstr. 20, 79085 Freiburg, Germany

## Abstract

**Background:**

Exposure to indoor air of private or public buildings contaminated with polychlorinated biphenyls (PCBs) has raised health concerns in long-term users. This exploratory neuropsychological group study investigated the potential adverse effects of chronic low-dose exposure to specific air-borne low chlorinated PCBs on well-being and behavioral measures in adult humans.

**Methods:**

Thirty employees exposed to indoor air contaminated with PCBs from elastic sealants in a school building were compared to 30 non-exposed controls matched for education and age, controlling for gender (age range 37–61 years). PCB exposure was verified by external exposure data and biological monitoring (PCB 28, 101, 138, 153, 180). Subjective complaints, learning and memory, executive function, and visual-spatial function was assessed by standardized neuropsychological testing. Since exposure status depended on the use of contaminated rooms, an objectively exposed subgroup (N = 16; PCB 28 = 0.20 μg/l; weighted exposure duration 17.9 ± 7 years) was identified and compared with 16 paired controls.

**Results:**

Blood analyses indicated a moderate exposure effect size (d) relative to expected background exposure for total PCB (4.45 ± 2.44 μg/l; d = 0.4). A significant exposure effect was found for the low chlorinated PCBs 28 (0.28 ± 0.25 μg/l; d = 1.5) and 101 (0.07 ± 0.09 μg/l; d = 0.7). Although no neuropsychological effects exceeded the adjusted significance level, estimation statistics showed elevated effect sizes for several variables. The objectively exposed subgroup showed a trend towards increased subjective attentional and emotional complaints (tiredness and slowing of practical activities, emotional state) as well as attenuated attentional performance (response shifting and alertness in a cued reaction task).

**Conclusion:**

Chronic inhalation of low chlorinated PCBs that involved elevated blood levels was associated with a subtle attenuation of emotional well-being and attentional function. Extended research is needed to replicate the potential long-term low PCB effects in a larger sample.

## Background

The neurobehavioral effects of polychlorinated biphenyls (PCBs) have been extensively studied in neonates and children [39,62,77]. However, no conclusive evidence is available on chronic nervous system effects in adult humans. The present neuropsychological group study explored the potential cognitive and affective consequences of long-term exposure to air-borne PCBs that were characterized by specific low chlorinated, ortho-substituted congeners. Effects sizes of behavioral and self-report measures were estimated to provide information that could be relevant for preparing extended epidemiological studies.

PCBs have been used as a component of insulation fluids, paints, and softening agents in lacquer, glues and sealing compounds. Low-level presence of PCBs has been discovered in many industrial settings in the USA and worldwide [14,57,58]. Due to the ubiquitous presence and poor degradation of PCBs, public health concerns continue to exist. Major exposure routes in humans include food intake, inhalation, and skin contact [59,72]. In particular, the indoor air of contaminated private or public buildings has been identified as a significant exposure source [29,65].

PCBs represent mixtures of up to 209 structurally related congeners differing by degree of chlorination which can be classified with respect to their similarity to 2,3,7,8-Tetrachlorodibenzo-p-dioxin (TCDD) [38,40,58,59]. Several of the congeners most frequently detected in the US population such as IUPAC-Nos. 138, 153, 180, have been categorized as nondioxin-like. Certain mono-ortho-substituted congeners, among them PCB 105, 118, and 156, represent the most frequently detected congeners with aryl hydrocarbon receptor activity (weakly dioxin-like). Several non-ortho-substituted congeners such as PCB 77, 126, 169 have been characterized as dioxin-like [14,38].

Animal experiments have stressed the neurotoxic potency of PCBs [25,72]. The mechanisms of PCB neurotoxicity appear to include direct cerebral effects as well as indirect steroid- and thyroid-agonistic modulation [30]. Changes in several neurotransmitter systems involving dopamine- and serotonin-antagonistic effects have been reported [45,46,48]. Perinatal exposure to nonplanar PCBs was associated with dopamine-antagonistic effects, whereas exposure to coplanar PCBs showed dopamine-agonistic results [10,66].

There has been a growing interest in the neurodevelopmental toxicity of PCBs [e.g., [39,71]]. Among the brain regions that have been studied for perinatal exposure in rats, the striatum, prefrontal cortex and cerebellum showed neurodevelopmental effects which also depended upon age and sex [25,48]. Prenatal exposure to low concentrations of mono-ortho substituted or coplanar congeners showed reduced LTP in the hippocampus [50]. Despite the fact that mono-ortho substituted and nonplanar PCBs have lower TCDD-toxicity equivalents (TEQ), some studies ascribed a greater neurotoxic potency to these substances [27,67].

In human subjects, a considerable body of research has reported negative associations between prenatal PCB exposure and cognitive functioning and motor development in childhood [62,77]. However, the information available on long-term neurobehavioral consequences in adults is sparse and conclusive results are not yet available. For example, acute PCB intoxication by contaminated food was associated with subjective complaints such as fatigue, headache, dizziness, muscle weakness and memory and concentration problems [15,16,56]. Consumption of PCB contaminated fish was associated with memory and learning impairment [61].

Contaminated indoor air has also been identified as a significant source of chronic PCB exposure. Potential long-term health effects in school and office buildings where elastic sealants containing technical PCB mixtures were used have raised public health concerns [7,12,29,42,49,65]. The potential neuropsychological effects of such long-term inhalation remain unknown.

The present study was initiated after low chlorinated PCBs were detected in indoor air of three school buildings and verified by biological monitoring in employees of one of these schools [29]. The latter group was subjected to a health-screening program including neurobehavioral testing. Due to the lack of conclusive neurobehavioral results in adult humans, the purpose of this study is exploratory. Behavioral effects were expected for executive, that is, frontostriatal function being modulated by potential dopamine-antagonistic effects. Firstly, we tested the global hypothesis that there is a difference between exposed subjects and controls. Secondly, estimation statistics were computed to obtain effect size information that might be useful for risk assessment and for evaluating the reproducibility independent of sample size.

## Methods

The present study was initiated after PCB-contaminated elastic sealant material was detected in a school building and indoor air concentrations of up to 10.655 ng/m^3 ^were measured. The school was closed for renovation and employees were immediately submitted to a surveillance procedure that also included neuropsychological testing. All subjects underwent a medical examination including history of medical and psychosocial life events, environmental risk factors and dietary habits. An identical procedure was carried out in matched controls employed by an uncontaminated secondary school.

### Study population

60 teachers and employees of two secondary modern schools were investigated. Thirty subjects were chronically exposed to air-borne PCBs in a school located in a rural region close to Heidelberg, Germany. This PCB group represents the total staff of this school. Thirty controls with no PCB exposure at work were drawn from another secondary school located in the city area of Heidelberg. The latter subjects were matched with the PCB group for education, age and professional status (see Table 1). The mean age was 49.2 years (SD = 7 years, range 37–61 years), with no differences between the PCB-group (48.2 years, SD = 7 years, range 39–60 years) and controls (49.9 years; SD = 7 years, range 37–61 years). The exposure group included 12 women whereas controls encompassed 18 women (χ^2^(1) = 1.67; p = .20). Gender differences of PCB levels that might be, for example, the result of excretion of PCBs during breast-feeding could not be confirmed [29]. Moreover, no substantial interactions of gender with exposure group were found for the present neurobehavioral variables. Nevertheless, the reported statistics given in the Tables were adjusted for the gender main effect to obtain unbiased information. Moreover, in a re-analysis of the data, an objectively exposed subgroup (> PCB 28 median 0.20 μg/l) was identified and compared with gender-matched controls.

**Table 1:** Demographic and exposure data of subjects exposed to PCB and of control subjects

	**PCB**		**Controls**				
	
	Mean	SD	Mean	SD	*F[1,56]*	*p*	
Gender [N; m/f]	18/12		12/18		1.67^1^	0.20	
Age [a]	48.2	7	49.9	7	0.89	0.35	
Education [a] ^2^	12.5	2	12.4	2	0.03	0.86	
Vocational index ^2^	5.9	0	5.8	0	0.39	0.54	
Estimated intelligence [IQ] ^2^	117.3	5	117.3	4	0.00	0.99	
Height [cm]	174	8	169	7	1.91^3^	0.17	
Weight [kg]	76	14	66	12	3.00^3^	0.09	
BMI [kg/m^2^]	24.7	3	23.5	3	2.27^3^	0.14	
*Self-reported data*							
Alcohol consumption [g/week]	94.4	76	72.4	75	1.26	0.27	
Nikotin consumption [cig./d]	6.4	10	5.7	10	0.07	0.79	
Q16-Score ^4^	4.4	4	3.6	4	0.67	0.42	
EQ Euroquest complaint score ^4^	146.4	32	131.6	31	3.29	0.07	
Memory and attention	27.0	6	23.9	6	3.62	0.06	
Drive and motivation	23.7	6	19.7	6	6.17	0.02	*
Tiredness	25.2	6	23.0	6	1.98	0.16	
Emotional reactivity	20.0	6	17.7	6	2.41	0.13	
Sensory complaints	8.1	3	7.4	3	0.65	0.42	
Motor complaints	5.2	2	4.8	2	0.96	0.33	
Cardiovascular complaints	14.8	5	12.0	5	4.76	0.03	*
Bowel and stomach	9.7	3	10.5	3	0.96	0.33	
Head and neck	11.0	4	12.3	4	1.90	0.17	
Stress inventory score ^4^	25.6	44	23.0	43	0.05	0.82	
*Exposure indices*							
PCB-28 [μg/l]	0.28	0.25	0.016 ^5^	0.02		0.0001	***
PCB-101 [μg/l]	0.07	0.09	0.01 ^5^	0		0.0003	***
PCB-138 [μg/l]	1.29	0.69	1.13	0.46	1.44	0.16	
PCB-153 [μg/l]	1.68	0.96	1.56	0.58	1.07	0.29	
PCB-180 [μg/l]	1.14	0.65	0.94	0.39	1.76	0.08	
Total PCBs [μg/l]	4.45	2.44	3.65	1.40	1.87	0.067	
Total occupational time [a]	20.9	6	22.0	9	1.09	0.28	
Duration of exposure [a] ^6^	16.7	9	n.a.				
Weighted duration of exposure [a] ^6,7^	10.5	6	n.a.				

^1^χ^2^[1]; *: p < 0.05; ***: p < 0.001.

^2 ^According to [76]

^3 ^Statistically adjusted for gender, F[1,55]

^4 ^A greater score corresponds to a greater number of complaints

^5 ^Median and IQR/2 is given, 95 percent < measurement threshold

^6 ^n.a.: not applicable

^7 ^Considering mean time of presence at school.

The profile of vocational activities of the two populations of employees was comparable (number of occupational years: PCB: 20.9 ± 6 years, controls: 22.0 ± 9 years; weekly working hours at school: PCB: 24.6 ± 6 h; controls: 24.3 ± 9 h). The exposed group spent 4.2 ± 4 years of their vocational life outside and 16.7 ± 7 years within the contaminated school building (range 1–25 years). Assuming 40 weeks working time per year, the mean weighted exposure duration was 10.5 ± 6 years.

According to their history, laboratory tests and a medical examination, pathological conditions of the nervous system could be ruled out. The clinical interview and self-report questionnaire yielded no history of neurological or psychiatric disorders. This lack of psychiatric diagnoses may be accounted for by the fact that persons with active disease are not permitted to remain in employment as teachers.

77% of all subjects did not take any medication; 5% of the total group reported taking drugs for allergies, hypertonia or hypothyreosis, but no drugs with substantial cerebral side effects. Physical measures were within normal limits (mean normative T-values for the body mass index (BMI): PCB: 54.9 ± 3.5; controls: 52.0 ± 5.0; n.s.) and an increase or decrease of body weight was not reported. Alcohol and nicotine consumption was moderate and did not differ between groups (Table 1).

### External exposure

Contamination by PCBs was determined by chemical analysis of indoor air and of elastic sealant materials. Air samples were collected during 24 h periods with closed doors and windows at a temperature of 20–22°C. External exposure was done by commercial institutes, analyzed according to standard procedures, and collected by the state Public Health Authority [for detailed data on indoor air PCB-concentrations of highly contaminated rooms, see [28,29]]. These analyses showed that the sealant material contained up to 50 percent of PCB. Indoor measurements revealed total airborne PCB concentrations of up to 17.460 ng/m^3^. Air concentrations in unrenovated rooms were between 2.870 ng/m^3 ^and 10.655 ng/m^3^.

In order to exclude other possible sources of exposure to chlorinated pollutants, subjects were interviewed concerning nutritional factors and life style. No differences were evident concerning wood interiors potentially treated with preservatives (PCB 50%; controls 40%; χ^2^(1) = 0.27; n.s.), leather wear PCB (60%; 47%; χ^2^(1) = 0.67; n.s.), or daily consumption of meat products (53%; 30%, χ^2^(1) = 2.47; p = .12). Consumption of fish was more frequent in controls (20%; 57%, χ^2^(1) = 10.15; p = 0.001). No previous occupations were mentioned that might indicate exposure to other toxic substances. In the PCB group, three persons might occasionally have had contact with chlorinated compounds (lab technician, joiner, plumber). One control subject had worked in a brewery, another as a lab technician. The number of chemistry teachers was comparable in both groups (PCB 33%, controls 20%). The total frequency of these potential vocational risk factors was not significantly different (PCB: 37%; controls 20%; χ^2^(1) = 1.31; n.s.). The confounding effects of additional exposure sources appear to be irrelevant (Table 2).

**Table 2:** Correlations of external exposure indices and internal PCB-values in all subjects

	**PCB 28**	**PCB 101**	**PCB 138**	**PCB 153**	**PCB 180**	**Total PCB**
Age^1^	0.17	0.17	0.27*	0.27*	0.26*	0.28*
BMI^1^	0.39**	0.26*				
Hours of work/week^1^	0.63***	0.51***	0.19		0.21	0.25^x^
PCB years of exposure^1^	0.71***	0.54***	0.37**	0.32*	0.39**	0.43***
Weighted total exposure index^1^	0.71***	0.59***	0.39**	0.33**	0.40**	0.45***
Alcohol/week^1^		0.31*	0.27*	0.24^x^		0.22^x^
Cigarettes/day^1^				-0.20	-0.26*	-0.17
Alternative vocational sources^2^				0.28*		0.18
Leather clothing^2^	0.24		0.24		0.33*	0.33*
Consumption of fish^2^	0.42***	0.27*				
Consumption of poultry^2^					0.19	

Note. *: p < 0.05, **: p < 0.01, ***: p < 0.001 nominal α; only correlations with at least a small effect size are given; correlations with potential exposure sources such as indoor installations of chemically treated wood, frequent meat or milk consumption were trivial and are not presented; correlations with large effect sizes are printed in bold.

^1 ^Spearman rank correlations

^2 ^Cramer-V.

### Biological Monitoring

Venous blood samples were drawn by the local Public Health Unit during a medical examination. Blood samples were analyzed by the state Public Health Authority using mass spectrometric gas chromatography (GC-MS) with standard protocols [see [29,65]]. Air-borne PCBs have previously been assessed by GC analyses of representative congeners such as PCB 28, 52, 101, 138, 153, and 180. These compounds have been used as markers of the specific exposure effects that can be traced back to polymer plasticizers used in Germany in the 1970's [3,29,37].

These congeners were analyzed according to the routine methods established by national authorities [37]. Although some authors recommended lipid standardization for the measurement of persistent lipophilic chemicals [e.g., [11]], a recent simulation study showed that PCB lipid standardization or the division of serum concentrations by serum lipids is potentially prone to bias [63]. Since group differences of serum lipids were not evident and because lipid adjustment is likely to produce spurious associations and biased results [63], unadjusted values were used.

A PCB sum value was computed except PCB 52 because quality assurance requirements failed for this congener [29]. A total toxicity index was not estimated because the TEQ concept is based on TCDD-toxicity equivalents mainly involving dioxin-like effects of coplanar PCBs. However, these congeners were not in the focus of the present study.

Blood sampling was carried out on average 4 weeks after the last exposure to contaminated air. The interval between first air sampling and blood sampling was 3 months. The interval between blood sampling and neuropsychological testing was 1 to 3 days. In addition to internal indicators, a weighted cumulative index was computed which indicated the total duration of exposure taking into account full- or part-time occupation and working days per year.

### Neurobehavioral assessment

Standardized neuropsychological testing [33,44] was used to assess subtle subjective and behavioral changes. Tests selection was motivated by previous findings in humans [61] and experimental animals [25,48,50]. Most of the tests used have been recommended by the WHO due to their known sensitivity to neurotoxic compounds [2] and have been integrated into current neurotoxicity batteries [13]. The battery is only briefly summarized here because it has been described in previous work [54,55].

In addition, computerized testing of attention was implemented with the Test battery for Attentional Performance (TAP) [78]. The TAP has been established in the context of an EU Biomed project for the standardized assessment of attention disorders in brain damaged patients [80]; its subtests are equivalent to the reaction tasks of current computerized neurotoxicity batteries such as the Milan Automated Neurobehavioral System (MANS) [13].

All neuropsychological investigations were performed in the morning using an uncontaminated environment. The administration of the neuropsychological battery took about 90 min including a 10 min break. The order of tests was randomized across subjects except for memory tests that required a fixed retention interval.

Since neuropsychological measures are partly intercorrelated, explorative factor analyses (principal component analyses with Varimax rotation, using data from a pool of available control subjects, N = 72) were computed separately for behavioral and self-report variables. The Scree-test suggested 8 factors for behavioral measures (each explaining 8 to 15 percent of the variance, total 80 percent) and 5 factors for self-report measures (each explaining 15 to 30 percent of the variance, total 70 percent). The obtained factor structure was used to group the scores and to derive factor descriptions (using variables with loads >.50). Moreover, median effect sizes were computed for each factor and presented in the Tables.

### Self-report measures

Subjective complaints and personality trait measures were organized in five clusters: current mood/emotional state, attention and motivation state, trait emotionality and health complaints, introversion, and sociability. Aggregated scores were also computed for each factor. In addition, psychosocial life stress was assessed [69] and weighted yielding a sum score for stressful events during the previous two years [36].

#### State descriptions of general physical well-being and mood

The questionnaire Q16 [35] is a well-known instrument for assessing neurotoxicity related symptom descriptions in solvent-exposed workers. Furthermore, a German version of a neurotoxicity symptoms questionnaire [17] was used to assess current complaints that are potentially related to neurotoxicity (items were aggregated according to the factorial structure of the Freiburger Beschwerdenliste (FBL-R) [23]).

#### Experience of attention and motivation state

State descriptions of attention and motivation as experienced in daily life were assessed by a 27-item Questionnaire of experienced deficits of attention (FEDA) [79], yielding scores for the factors motivation and drive, fatigue and slowing of practical activities, and distractibility of mental processes.

#### Trait measures of general physical well-being and emotional instability

The Freiburg Personality Inventory (FPI-R) [24] includes the scales impaired well-being (FPI Factor SI), aggressive arousability (FPI Factor SII), poor satisfaction with life (FPI 1), arousability (FPI 5), emotional stress (FPI 7), physical complaints (FPI 8), health worry (FPI 9), and emotional instability (FPI N). Depressed affect during the previous week was assessed by the Center for Epidemiological Studies Depression Scale (CES-D), German version (ADS) [34]. Other scales loading on this factor were General health complaints as assessed with the FBL [23] which contains 10 complaint item clusters such as the sum of bodily complaints (FBL11), general well-being and physical complaints (FBL1), emotional reactivity (FBL2), cardiovascular complaints (FBL3), bowels and stomach (FBL4), tension and strain (FBL6), sensory sensitiveness (FBL7), pain (FBL8), and skin problems and cold hands (FBL10).

#### Introversion

This factor included the scales introversion (FPIE), low aggression (FPI6), reserve and low openness (FPI10), as well as low achievement and work motivation (FPI3).

#### Sociability

This sociability/psychoticism factor included inhibition (FPI4), low social orientation (FPI2), motor restlessness (FBL9), and head-neck irritation (FBL5).

### Behavioral tests

#### General intelligence

An estimation of present intelligence (IQ) as an overall measure of intellectual functioning was derived from the information, similarities, block design, and picture completion subtests of the Wechsler Adult Intelligence Scale (WAIS) [20,70].

#### Fluid intelligence

This factor included fluid intelligence measures related to verbal concept formation and reasoning processes (WAIS similarities, picture completion, and digit span forward).

#### Visuo-motor performance

Visuo-motor performance was assessed by the WAIS Block design subtest.

#### Concentration, alertness and speed

Selective attention and exploration speed was assessed with the Trail Making Test parts A and B (seconds) [44]. Alertness was measured with a simple and a cued reaction time task from the TAP [78]. Subjects were requested to respond whenever a cross appeared on the screen. In one condition, 40 visual stimuli were presented, each preceded by an acoustic warning stimulus. In the other condition, the cross appeared without warning. The difference between simple and cued reaction time was used as a measure of phasic alertness.

#### Working memory

This factor included the visual span forward and backward, verbal span backward subtests taken from the Wechsler Memory Scale-Revised (WMS-R) [32,74]. The digit symbol subtest from the WAIS was used to assess working memory, flexibility and speed [70]. Moreover, the TAP-subtest error scores for working memory, response shifting, and divided attention were associated with this factor [78]. The *working memory *subtest required a continuous control of the information flow through short-term memory. One-digit consecutively presented numbers had to be compared continuously with the preceding-but-one number (N-back task). In the *response flexibility *task, shifting of focused attention was tested by alternations between two sets of targets (letters or numbers) that were presented simultaneously and randomly, one on the left, the other on the right side of the fixation point. From one presentation to the next the target changed from letter to number and vice versa. The subject was requested to press the key on the side of the target (left or right). *Divided attention *was investigated with a dual task paradigm which was realized by a simultaneous visual/acoustic choice condition. A series of 75 matrices was presented on the screen, each for a duration of 3 s, with an inter-stimulus interval of 500 ms. A matrix consisted of a regular array of 4 × 4 dots with seven small 'x's superimposed randomly upon them. The subject was required to react whenever four 'x's formed a square. Simultaneously, the subjects listened to high and low pitched tones in regular alternation for a period of 5 min. Occasionally, a tone was followed by a tone of the same frequency that had to be detected.

#### Learning and memory

Verbal memory tests included the WMS-R subtest logical memory (immediate and delayed recall of stories). Visual memory scores were derived from the WMS-R visual reproductions (immediate and delayed recall of designs) [32]. Additionally, the Auditory Verbal Learning Test (AVLT) was used to assess free recall from verbal short-term and long-term memory [44].

#### Specific frontal lobe functions

Word fluency measures were obtained from the Regensburg Word Fluency Test (RWT) [1] and a design fluency task (production of non-recurrent figures) was added.

#### Psychomotor speed and attention

The alertness, working memory, response shifting, and divided attention subtests from the TAP [78] were used to assess simple and complex choice reaction time.

### Statistical analysis

A traditional strategy for risk assessment in populations is to apply distribution-based statistics. However, it could also be useful to express exposure-related performance differences in a metric-free form [6]. Since the distribution-based null hypothesis testing approach (NHT) critically depends upon sample size, this does not provide information as to whether an effect is potentially replicable in larger study groups [31]. An estimation statistics approach is suitable to quantify group differences by means of the effect size d [18,31]. Therefore, a dual approach was applied in the present study [52,53]:

First, the question whether a behavioral PCB effect can be demonstrated was answered by NHT. ANOVAs with the factors exposure group and gender were computed to test the global hypothesis of μ_PCB_<μ_CON_. The results of the univariate F-tests for the exposure effect (with means adjusted for gender) are provided in the Tables. The α-level was set to p = .10 in order to control for the β-error (since it is inappropriate not to detect subtle differences at this stage of research [75]). Nominal α's are reported in the Tables. Since MANOVAs could not be computed due to insufficient samplesize [8], a revised Bonferoni α-adjustment was used for correction of dependencies [19]. Moreover, subjects with blood values above the PCB 28 median were compared with matched control subjects in a reanalysis of the data.

Second, estimation statistics were computed to evaluate which behavioral effects might be replicable independent of sample size [18]. Effect sizes of these differences were derived from η^2 ^of the gender adjusted group effect (d_1_, corresponding to the above NHT approach). An additional effect size estimate was computed as the deviation of the empirical T-score of a behavioral variable from the distribution of the normative sample (d_2_, corresponding to the specific one-tailed hypothesis of μ_T(PCB)_<50).

All reported d- and T-values were uniformly scaled so that elevated values of self-report-variables (d≥0.20, T>50) indicated elevated scores or complaints, whereas lower values in behavioral tests (d≤-0.20, T<50) indicated attenuated performance. An effect size for a behavioral measure was classified as meaningful in terms of potential reproducibility and marked with Δ in the Tables if both d_1 _as well as d_2 _showed the predicted attenuation at least to a moderate degree (e.g., d≤-0.20) [18].

To determine dose-response relationships, we computed correlations between exposure variables (total PCB, the marker congener PCB 28 [2,4,4'-trichlorobiphenyl], and weighted exposure duration) and age-adjusted variables in the exposed group. Positive correlations with exposure were expected for self-report variables and inverse associations were expected for behavioral variables. Depending on distribution characteristics, the results were verified using Spearman rank correlations. To control for spurious correlations and potential suppressor effects, the correlation analyses were done with and without partialling out specific confounders. Since self-reported complaints may be influenced by the subjects' openness (PCB subjects were more reserved, see Table 3) and by alcohol consumption (PCB subjects showed slightly elevated values compared to controls, see Table 1), these variables were considered in the analyses. Since behavioral test performance may be confounded by exposure-independent intelligence level, an estimate of this variable as well as alcohol consumption was considered. Analyses were done with MS-Excel, SPSS [68] and SAS for Windows [60].

**Table 3:** Mood, physical complaints and personality trait measures: primary and aggregated secondary factors in 30 PCB-exposed and 30 control subjects

	**PCB**		**Controls**		**Effect sizes**				
	
	Mean	SD	Mean	SD	F[1,56]^2^	p^2^	d_1_^3^	d_2_^3^	
*Current emotional mood state^4^*							*0.41^5^*	*0.36^5^*	
EQ Tiredness/deactivation	54.3	13	50	10	2.06	0.16	0.38	0.36	Δ
EQ Emotional reactivity	53.6	11	50	10	2.41	0.13	0.41	0.33	Δ
EQ Low well-being	55.5	13	50	10	3.29	0.07	0.49	0.47	Δ
*Attentional and motivational state*							*0.25*	*0.24*	
FEDA Poor motivation and drive	50.4	12	48.2	11	0.55	0.46			
FEDA Fatigue and slowing	52.5	11	49.7	10	1.07	0.30	0.28	0.24	Δ
FEDA Distractability	55.0	11	48.8	10	5.26	0.03	0.61	0.48	Δ
*General physical well being and emotional instability (trait)*							*0.02*	*0.21*	
FPI Factor SI/impaired well-being	48.1	6	50.0	6	1.40	0.24	-0.32	-0.23	Δ
FPI Factor SII/aggr. arousability	48.3	7	52.3	7	4.44	0.04	-0.57		
FPI 1 Poor satisfaction with life	46.6	10	49.6	10	1.35	0.25	-0.31	-0.34	Δ
FPI 5 Arousability	50.5	12	51.8	12	0.16	0.69			
FPI 7 Emotional stress	51.9	10	51.6	10	0.01	0.92			
FPI 8 Physical complaints	46.2	9	47.3	9	0.23	0.64		-0.40	
FPI 9 Health worry	47.7	9	51.5	9	2.39	0.13	-0.42	-0.23	Δ
FPI N Emotional instability	48.4	11	47.2	11	0.16	0.69			
FBL11 Sum of bodily complaints	54.6	9	53.4	9	0.25	0.62		0.48	
FBL 1 Well-being/phys. complaints	53.9	9	49.8	9	3.22	0.08	0.48	0.41	Δ
FBL 2 Emotional reactivity	55.0	10	51.9	9	1.65	0.20	0.34	0.51	Δ
FBL 3 Cardiovascular complaints	53.1	9	52.3	9	0.12	0.73		0.32	
FBL 4 Bowels and stomach	53.8	8	53.9	8	0.00	0.96		0.41	
FBL 6 Tension, strain	53.2	10	52.2	10	0.17	0.68		0.32	
FBL 7 Sensory sensitiveness	56.0	9	55.2	9	0.13	0.72		0.63	
FBL 8 Pain	52.3	10	51.9	9	0.03	0.87		0.23	
FBL 10 Skin and cold hands	54.2	10	55.9	10	0.44	0.51		0.42	
ADS Depressed affect	48.0	9	47.8	12	0.01	0.94			
*Introversion*							*0.45*	*0.35*	
FPI E Introversion	54.6	11	50.4	11	2.12	0.15	0.39	0.43	Δ
FPI 6 Low Aggressivity	54.7	8	49.2	8	6.42	0.01	0.68	0.51	Δ
FPI 10 Reserve/low openness	50.9	11	45.7	10	3.63	0.06	0.51		
FPI 3 Low achievement/work motiv.	52.6	9	52.5	9	0.00	0.97		0.27	
*Sociability*							*0.38*	*0.34*	
FPI4 Inhibition	52.0	11	47.9	11	2.22	0.14	0.40		
FPI 2 Low social orientation	42.9	8	47.5	8	4.45	0.04	-0.57	-0.77	Δ
FBL 9 Motor restlessness	54.7	9	51.4	9	1.84	0.18	0.36	0.48	Δ
FBL 5 Head-neck irritation	56.1	9	51.5	9	3.73	0.06	0.52	0.63	Δ
*Aggregated secondary factors*							*0.30*	*0.26*	
Current mood/emotional state	53.6	8	50.4	8	2.25	0.14	0.40	0.40	Δ
Reduced attention and motivation	52.6	10	49.7	10	1.25	0.26	0.30	0.26	Δ
Low well being/trait emotionality	49.3	7	50.4	7	0.42	0.52			
Introversion	53.2	6	49.5	6	5.08	0.03	0.61	0.38	Δ
Low Sociability	47.4	6	47.7	6	0.02	0.88		-0.31	

^1 ^T-Score; greater values correspond to elevated feature score

^2 ^F-values for group with control of gender, nominal α

^3 ^Effect sizes in comparison with controls (*d_1_*; with control of gender) and in comparison with normative sample (*d_2_*); Δ: at least moderate effect size of d_1 _and d_2 _suggesting potential reproducibility

^4 ^T-values relative to controls

^5 ^Effect size median.

## Results

### External exposure

**Figure 1:**
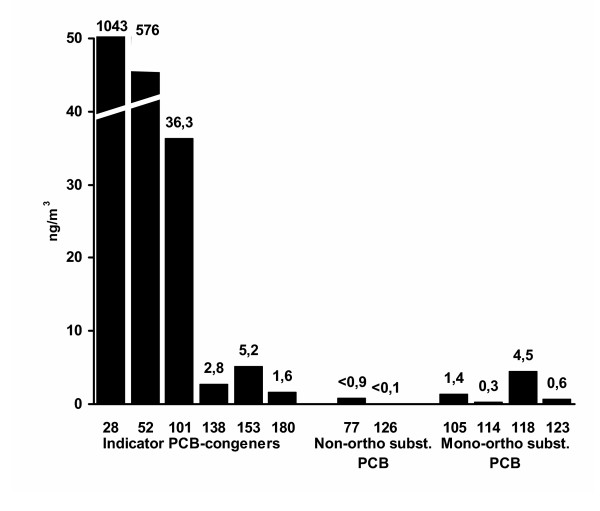
**PCB-concentrations of indoor air**. Means of PCB measurements from three highly contaminated rooms (room 303, 407 and teacher's room) [from 29, p. 1058, Table 3, modified].

External exposure measurements indicated that 5 rooms were contaminated with indoor air PCB values ranging from 1.587 to 10.655 ng/m^3 ^(mean 7.749 ng/m^3^) [28,29]. The elastic sealant material was the primary source of exposure but walls and floors showed a similar PCB pattern. The lower chlorinated congeners 28 and 52 were responsible for about 90% of measured PCB marker congeners. The higher chlorinated and non-ortho-substituted or mono-ortho-substituted PCBs were of minor importance [29]. Figure 1 shows aggregated exposure measures for contaminated rooms indicating increased PCB values for the congeners 28, 52 and 101. The school was closed and renovated; follow up measurements in renovated rooms indicated that PCB contamination had fallen below 3.000 ng/m^3^.

### Internal exposure

**Figure 2:**
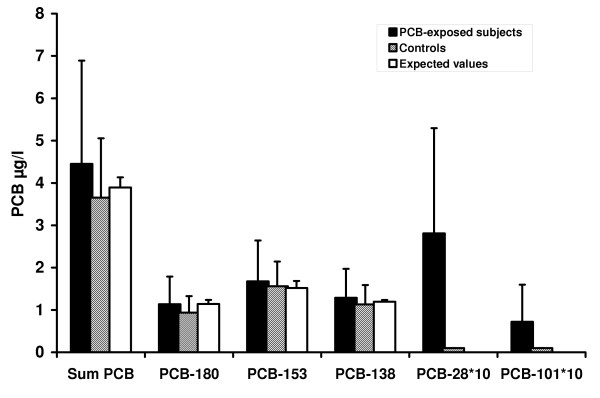
**Blood values of PCB-exposed and control subjects**. Blood values (means and standard deviations) of the PCB-exposed subjects and the control group (median PCB-28 and -101 levels were below 0.01 μg/l in controls). Expected values (background exposure) were estimated for each exposed subject from age-group related reference values [median PCB-plasma values for PCB-138, -153 and -180 according to 37].

Overall PCB exposure had a low to moderate effect size (d = 0.4 – 0.5) relative to expected background exposure values as derived from individual, age-group related median plasma PCB levels taken from national exposure data [5,37]. This was mainly due to low chlorinated PCBs (PCB 28: 0.28 ± 0.25 μg/l; PCB 101: 0.07 ± 0.09 μg/l), which are known to accumulate from respiratory rather than from nutritional sources (Figure 2). In contrast, exposure to the congeners PCB 138, 153 and 180 was not markedly elevated and most likely associated with general background exposure including food. More than 90 percent of control subjects showed PCB 28 and PCB 101 levels below detection threshold, whereas most of the exposed subjects showed detectable blood levels of these congeners (p < 0.001, Fisher's exact test; see Table 1). PCB28 values were above the range of controls in 53 percent of the PCB-exposed subjects corresponding to a large effect size (d = 1.5).

Table 2 shows the relationship of internal and external indices. Blood PCB 28 and 101 were correlated with the cumulative index on an adjusted significance level. For PCB 138, 153 and 180, these correlations were not significant. Above-average fish consumption was also correlated with PCB 28. Therefore, we examined whether PCB 28 could have been modified by alternative sources of exposure. An explorative logistic regression model with stepwise variable selection was used which included demographic information as well as potential sources of exposure. A significant model for predicting blood values (which were dichotomized at the median of controls) could only be generated for PCB 28 (χ^2^(2) = 59.5; p < 0.0001; R^2 ^= .84). The weighted exposure index significantly contributed to the explanation of variance (p = 0.005). Age, alcohol and nicotine, as well as fish consumption had no strong predictive value. A similar but weaker model was found for PCB 101 (χ^2^(1) = 19.6; p < 0.0001; R^2 ^= .42).

Thus, nutritional and other exposure sources appeared to be of minor importance for predicting the present PCB 28 blood values. In contrast, all other congener values were associated with additional risk factors such as leatherwear, additional vocational sources or potentially contaminated food, etc. This confirms the decision to focus dose-response analyses on PCB 28 which was causally connected with the present elastic sealants material.

### General health

General health of all subjects was satisfactory and no symptoms indicated acute or chronic PCB intoxication. The extent of complaints as assessed by self-report inventories did not exceed expected values. Diseases reported most frequently by both groups were allergies (42%), asthma and bronchitis (29%), and hypertonia (16%). No significant group or gender related differences were evident for complaints associated with the cardiovascular system (PCB: 10%; controls: 20%), skeletal motor-system (10%; 17%), respiratory system (17%; 17%), allergies (27%; 23%), thyroid dysfunction (7%; 3%), hepatitis (7%; 3%), diabetes (0%; 7%), or headache (0%; 7%). Neurological or psychiatric disorders could not be identified.

### Neuropsychological results

Whereas the Q16 did not reveal elevated complaints, the Euroquest indicated a trend towards low motivation and cardiovascular problems (Table 1) as well as reduced well-being and distractibility of mental processes (Table 3). Moreover, estimation statistics yielded moderate effect sizes for distractibility, low level of well-being, head-neck pain syndrome, and for the personality trait variables of introversion, social orientation and low aggression.

The behavioral results indicated comparable intellectual functioning in both groups (Table 4). Both teachers groups showed normative values and effects sizes suggesting a high level of verbal intelligence (d_2_>1). Small, yet non-significant effects corresponding to hypothesis were found only for the TAP divided attention subtest. Moderate effect sizes were also observed for phasic alertness and Trails A. A general trend towards slightly increased reaction times in all of the computerized attention tasks in the exposed group must be noted which, however, were within the range of the normative population. Learning and memory performance of both groups was average. Inconsistent with hypothesis, the exposed group showed better immediate and delayed visual memory performance; however, this effect could not be replicated by the re-analyses summarized below.

**Table 4:** Neuropsychological results in 30 PCB-exposed and 30 control subjects

	**Raw Values**				**T-Scores^1^**								
	
	**PCB**		**Contr.**		**PCB**		**Contr.**		**Effect sizes**				
	
	Mean	SD	Mean	SD	Mean	SD	Mean	SD	F[1,56]^2 ^p^2^		d_1_^3^	d_2_^3^	
*General intelligence*											*-0.03^4^*	*1.09^4^*	
WAIS IQ	72.2	9	72.2	9	113.3	10	115.8	10	0.86	0.36	-0.25	1.09	
WAIS General knowledge	18.2	3	17.7	3	58.2	9	58.8	9	0.07	0.79		0.97	
WMS Digit span forward	9.7	2	9.2	2	60.0	10	57.7	10	0.79	0.38	0.24	0.99	
WAIS Similarities	19.7	3	19.3	3	63.0	9	62.8	9	0.01	0.94		1.38	
WAIS Picture completion	12.4	2	12.7	2	61.1	10	63.2	10	0.74	0.39	-0.23	1.15	
*Visuo-motor performance*											*-0.21*	*0.47*	
WAIS Block design	21.9	6	22.5	6	53.6	7	55.0	7	0.63	0.43	-0.21	0.47	
*Concentration, alertness and speed*											*-0.41*	*0.05*	
Trail making Test, part A	37.5	11	34.4	11	50.1	7	53.5	7	3.67	0.06	-0.51		
Trail making Test, part B	79.3	27	74.6	27	53.7	7	55.6	7	1.11	0.30	-0.28	0.46	
TAP Phasic alertness	0.024	0.1	0.058	0.1	45.9	12	50.8	12	2.34	0.13	-0.41	-0.38	Δ
*Working memory*											*-0.08*	*0.08*	
WMS Visual span forward	8.5	2	8.2	2	50.7	13	50.5	13	0.00	0.95			
WMS Visual span backward	8.0	2	7.3	2	50.6	10	47.6	10	1.31	0.26	0.31		
WMS Verbal span backward	7.2	2	7.4	2	50.2	11	52.8	11	0.84	0.36	-0.25		
TAP Working memory/errors	4.9	4	6.3	4	52.3^5^	11	50^5^	10	2.08	0.16	0.39	0.23	
TAP Response shifting/errors	6.3	4	6.0	4	47.5^5^	14	50^5^	10	0.09	0.76		-0.20	
TAP Divided attention/errors	2.0	1	1.2	1	43.5^5^	10	50^5^	10	4.79	0.03	-0.58	-0.65	Δ
WAIS Digit symbol	53.3	11	54.0	11	56.3	9	57.1	9	0.12	0.73		0.61	
*Verbal memory*											*0.40*	*-0.83*	
WMS Logical memory recall	22.5	7	21.7	7	42.2	9	41.5	9	0.09	0.76		-0.85	
WMS Logical memory delay	18.9	7	16.3	6	42.8	9	39.4	9	2.24	0.14	0.40	-0.83	
AVLT Word list learning	75.8	11	70.8	11	53.8	9	49.6	9	3.08	0.08	0.47	0.29	
*Visual memory*											*0.93*	*0.83*	
WMS Visual memory recall	39.5	5	34.2	5	61.7	10	52.4	10	13.26	0.001	0.97	1.24	
WMS Visual memory delay	34.2	9	27.1	9	56.3	12	46.2	12	11.09	0.002	0.89	0.43	
*Frontal lobe functions*											*0.24*	*0.42*	
RWT Word fluency	17.2	3	16.0	3	55.3	6	53.7	6	1.19	0.28	0.29	0.69	
Design fluency	30.6	7	29.3	7	51.5	9	50.2	10	0.53	0.47			
*Psychomotor speed [TAP, 78]*											*-0.39*	*-0.24*	
Simple reaction time (RT)	281.1	125	253.8	123	48.7	14	52.3	13	1.06	0.31	-0.27		
RT with warning stimulus	275.5	120	236.2	118	46.6	13	51.2	12	2.08	0.15	-0.39	-0.24	Δ
RT working memory task	687.7	229	556.5	226	46.2	12	53.5	12	5.91	0.02	-0.65	-0.36	Δ
RT response shifting task	869.9	275	847.3	270	48.0	12	52.3	12	2.06	0.16	-0.38		
RT divided attention task	702.5	96	665.8	94	42.8	10	46.8	10	2.56	0.12	-0.43	-0.69	Δ

^1 ^Lower values indicate poorer performance

^2 ^F-value for group effect adjusted for gender, nominal α

^3 ^Effect sizes (>0.2): *d_1 _*relative to controls, adjusted for gender; *d_2 _*relative to the normative population; Δ: at least moderate attenuation of d_1 _and d_2 _suggesting potential reproducibility

^4 ^Effect size median

^5 ^Relative to controls.

### Dose-response-relationships

Significant relationships of dose indicators (total PCB, PCB 28 and cumulative index) and response measures (self report or behavior) could not be demonstrated on an adjusted significance level. Self-reported complaints and mood state showed no substantial positive association with PCB or the cumulative index. For behavioral variables, however, several correlations were found for PCB 28. These correlations with figural fluency (r = -0.54; p < 0.01), simple reaction time (r = 0.31; p < 0.05), TAP response shifting errors (r = -0.31; p < 0.05), AVLT word list learning (r = -0.38; p < 0.05), and digit symbol (r = -0.32; p < 0.05) remained when rank correlations were computed or when estimated intelligence and alcohol were partialled out. Mood and personality variables showed no clear association with the behavioral data.

### Re-analysis of subgroup with elevated PCB 28 blood levels

Since exposure status was variable due to different working habits in contaminated rooms, a re-analysis was done with objectively exposed subjects with PCB 28 levels ≥ 0.20 μg/l. This congener was chosen because it was significantly elevated in the present sample and correlated with the indoor air PCB burden. Two persons from the former exposure group were assigned to the control group because they had been working in the contaminated school only for a short while and showed no elevated blood values. This objectively exposed group (12 males, 4 females, 49.8 ± 6 years, weighted exposure duration 17.9 ± 7 years, range 4–25 years) and controls (12 males, 4 females, 48.6 ± 8 years) were matched for sex, age and education and were comparable with respect to physical characteristics, alcohol and smoking. Estimated intelligence (IQ 119.5 ± 5 and 118.0 ± 7; d = 0.24) was partialled out in the analyses of behavioral data.

PCB 28 levels of exposed subjects (median = 0.30 μg/l; range 0.20–1.05 μg/l) were above the distribution of controls (> 0.01 μg/l). Significant differences were also found for PCB 138 (1.453 ± 0.59 μg/l and 0.953 ± 0.37 μg/l; p = 0.01), PCB 153 (1.906 ± 0.85 μg/l and 1.272 ± 0.47 μg/l; p = 0.01) as well as PCB 180 (1.316 ± 0.69 μg/l and 0.725 ± 0.25 μg/l; p = 0.003).

The comparison of neuropsychological data did not show differences on an adjusted significance level. Nevertheless, when the effect sizes for the five self-report factors were inspected, the aggregated value for attention/motivation showed a medium effect (mean T = 54.6 ± 10 and T = 47.8 ± 11; d = 0.58) that was due to greater report of tiredness and slowing (T = 54.6 ± 10 and T = 45.9 ± 7; d = 0.70). The exposed subgroup also showed a trend towards more frequent reports of emotional reactions (mean T = 53.1 ± 11 and T = 46.8 ± 10; d = 0.46). Openness to answer questionnaires correctly was similar in both groups. Relative to the total PCB group, the PCB exposure subgroup described greater inattention, tiredness, distractibility as well as emotional and aggressive reactions. For behavioral measures, the subgroup comparisons showed relevant effect sizes only for attentional functions as indicated by TAP phasic alertness (T = 45.3 ± 12 and T = 48.1 ± 14; d = -0.32) and response shifting (T = 45.2 ± 15 and T = 50.7 ± 9; d = -0.40).

### Re-analysis of subgroup with low PCB 28 blood levels

The subjects were aware of the fact that they had been exposed. The examiner was blind to the objective exposure status but not blind to the exposure site. Thus, the current study could only be performed in an open fashion. To test the hypothesis that prior information might have induced additional complaints or behavioral changes, 10 subjects with low objective PCB exposure (PCB 28≤0.1 μg/l) were identified and compared with matched controls. Although results were not significant on an adjusted level, there was a trend towards greater emotionality (T = 53.0 ± 9 and T = 43.5 ± 7; d = 1.1) and subjective distractibility (T = 59.9 ± 13 and T = 47.4 ± 5; d = 1.3) in the low PCB group. However, behavioral data showed no difference except of superior short term retention of visual designs (T = 62.5 ± 6 and T = 54.9 ± 8; d = 1).

## Discussion

Chronic low-dose inhalation of polychlorinated hydrocarbons has repeatedly raised health concerns in subjects exposed in their everyday work environment or at home. This study contributes to our knowledge of potential neurobehavioral effects in the area of chronic exposure to low chlorinated PCB congeners in adult humans.

### External and internal PCB exposure

Elevated PCB values were confirmed for low chlorinated congeners such as PCB 28, thus corresponding to previous reports [29,65]. This additional PCB burden relative to background exposure has been estimated to 2.8% [29]. This corresponds to an approximate PCB 28 exposure effect size of d = 0.34 or of d = 0.54 if PCB 52 is included. The present analysis yielded a large exposure effect size for PCB 28 (d>1) suggesting an acceptable discrimination from background exposure for this congener.

Although a significant increase was not observed for PCB 138, 153 and 180 relative to controls and to background exposure (Figure 2), significantly elevated levels were found for these congeners in the highly exposed subgroup. The fact that, for example, PCB 180 was found in the contaminated air (Figure 1) and was correlated with exposure duration but not with age could be a result of local low incorporation or of a slower rate of metabolization. The fact that PCB 180 was below background level in controls might have further added to this subgroup difference.

However, the measurement of low chlorinated compounds could be compromised by several factors. PCB 28, 52 and 101 show a relatively fast decomposition and half-life of about 60 days [29,64]. Due to the interval of 4 weeks between termination of exposure and blood sampling, these levels might have been underestimated. A decline of PCB blood values was observed in single subjects eight months after termination of exposure (data not presented).

Moreover, fluctuations of internal exposure due to factors such as individual differences in metabolism, room use and ventilation conditions may have further obscured dose-response relationships. Blood PCB values may also misrepresent the concentration in the brain where only about 10% of the blood PCB burden can be found [4]. Nevertheless, the fact that the cumulative exposure index successfully predicted internal PCB 28 suggests that the latter congener represents an appropriate marker of the present PCB burden.

PCB 28 is planar and mono-ortho-substituted but it can be regarded as nondioxin-like due to a low number of chlorine atoms. In contrast, neurobehavioral changes could also be mediated by dioxin-related toxicity effects of high-chlorinated and dioxin-like PCBs or by polychlorinated dibenzodioxins and -furans (PCDD/Fs) [10] which were not assessed in the present study. However, a pooled analysis of the current blood samples showed no significant increase of the planar PCB 77, 126 and 169 as well as dioxins [65] suggesting that our results are unlikely to be biased by dioxin-related toxicity.

Thus, a generalization of the present findings is limited to conditions of air-borne exposure to and confirmed incorporation of low to medium chlorinated PCBs. Since employees of other contaminated schools showed no elevated blood values for both low and high chlorinated congeners [12,22], an extrapolation to conditions without confirmed internal exposure does not seem warranted.

### Subjective and behavioral effects of chronic exposure to low chlorinated PCBs

The unadjusted statistics given in the Tables must be cautiously interpreted due to alpha inflation and multiple testing. On an adjusted significance level of p = 0.004, the global null hypothesis of at least one group difference in self report or neurobehavioral variables could not be rejected because differences between exposed and non-exposed subjects were relatively small. Additional estimation statistics were used to detect subtle effects and to evaluate the reproducibility independent of sample size. Moderate effect sizes were found for distractibility, well-being, as well as for trait measures indicating low aggression and greater social orientation. The results of subgroup analyses confirmed a trend towards increased self-reported tiredness and slowing, and emotional reactions.

However, awareness of the current PCB exposure condition might have induced stress in terms of fear of being intoxicated, attention towards complaints, or general behavioral activation. It has been shown, for example, that perceived olfactory stimuli may influence well-being, emotional reactivity and cause health concerns [21,51]. Certain personality traits may sensitize for the consequences of exposure events [43]. Self-report data could thus be confounded by prior information about exposure and health concerns. In our subjects, however, personality disorders or chemical sensitivities could not be ascertained. Nevertheless, the degree of complaints was not correlated with PCB exposure level and subjects with low PCB exposure also reported elevated complaints. Therefore, the present self-reports are likely to have been biased by prior information about exposure.

Behavioral measures, in contrast, are less likely to be influenced by such information. Weak to moderate effect sizes were found for attention measures such as alertness and response shifting. Furthermore, moderate correlations between PCB and behavioral variables were found for figural fluency, response shifting and digit symbol. These findings deviate from prior reports of reduced learning and memory but not executive function [61]. A possible reason might be a greater exposure of fish eaters to high chlorinated, mono-ortho or coplanar congeners. Such an elevation of nutritional PCBs could not be confirmed for PCB or control subjects reporting greater consumption of fish.

Certain chlorinated hydrocarbons appear to alter frontostriatal function by dopamine depletion in animals. For example, nonplanar PCBs produced dopamine-antagonistic effects in the striatum and prefrontal cortex [10,46,48,66]. In human subjects, emotional regulation, motivation as well as alertness and response shifting rest upon and require contributions of the ventromedial and superior prefrontal cortex [26,41]. It is well accepted that mental flexibility, shifting the attentional focus and other executive functions are associated with dopamine-related frontostriatal activity in healthy individuals [73]. Conversely, disorders of frontal brain regions may affect attentional functions, alertness and working memory [47] and mood [9]. The present subjective and behavioral effects are therefore compatible with the hypothesis of a subtle attenuation of frontostriatal functions.

The fact that the low exposure subgroup showed a trend of greater visual memory (in addition to elevated emotionality discussed above) suggests that additional confounders independent of exposure might have moderated behavioral performance. Given that schools tend to differ with respect to educational programs, teachers may therefore show similar performance differences. Nevertheless, group differences in teaching preferences, medical and psychosocial events, additional environmental risk factors or dietary habits could not be substantiated.

Taken together, the present estimation statistics provided continuous indicators of exposure [6] yielding low response effects in a relatively small sample. Despite the limitations of this exploratory approach, these results suggest that findings may be replicable and should be replicated in the context of a more comprehensive epidemiological study. A sample size of approximately 200 to 500 exposed persons would be necessary to produce "significant" results according to conventional statistics. Moreover, we have argued elsewhere that even low behavioral effects may provide soft endpoints of neurotoxicity that could incur considerable economical costs (loss of work efficiency or motivation) when a large population is affected across an extended time span [53].

## Conclusion

This exploratory neuropsychological group study showed that a discriminative, low chlorinated PCB marker congener typical for the present indoor air exposure condition could be identified. Although neurobehavioral effects could not be demonstrated by traditional significance testing, estimation statistics showed group differences with moderate effect sizes indicative of subjective attentional and emotional complaints as well as attenuated attention performance. Extended epidemiological research is needed to replicate and further substantiate the hypothesis of a subtle frontostriatal dysfunction in PCB exposed adults.

## Abbreviations

AVLT Auditory Verbal Learning Test

BMI Body Mass Index

CES-D Center for Epidemiological Studies Depression Scale (Allgemeine Depressions-Skala, ADS)

d Effect size measure (weak: d<0.2; moderate: d = 0.5; strong: d>0.8)

Δ Potentially relevant effect sizes d>0.2

EQ Euroquest Symptom Questionnaire

ETA^2 ^(η^2^); f Effect sizes derived from analysis of variance

FBL-R Freiburg Complaint Questionnaire Revised (Freiburger Beschwerdenliste)

FEDA Questionnaire of experienced deficits of attention (Fragebogen erlebter Defizite der Aufmerksamkeit)

FPI-R Freiburg Personality Inventory Revised (Freiburger Persönlichkeitsinventar)

MANS Milan Automated Neurobehavioral System

NHT Null hypothesis testing

PCBs Polychlorinated biphenyls

PCB 28 2,4,4'-Trichlorobiphenyl

PCB 52 2,2',5,5'-Tetrachlorobiphenyl

PCB 101 2,2'4,5,5'-Pentachlorobiphenyl

PCB 138 2,2',3,4,4',5'-Hexachlorobiphenyl

PCB 153 2,2',4,4',5,5'-Hexachlorobiphenyl

PCB 180 2,2',3,4,4',5,5'-Heptachlorobiphenyl

PCDD/F Polychlorinated dibenzodioxin/furan

Q16 Neurotoxicity Symptom Questionnaire

RT Reaction time

RWT Regensburg Word Fluency Test

T T-Score (normative values with mean = 50, standard deviation = 10)

TAP Test Battery for Attentional Performance

TCDD 2,3,7,8-Tetrachlorodibenzo-p-dioxin

TEQ Toxicity Equivalents

IQ Intelligence Score (values with mean = 100, standard deviation = 15)

WAIS Wechsler Adult Intelligence Scale

WMS-R Wechsler Memory Scale Revised

## Competing interests

The author(s) declare that they have no competing interests.

## Authors' contributions

MP provided all basic contributions to conception, design and methodology, performed the statistical analysis, wrote the draft version of the article and revised it critically for content. MK participated in the design and coordination, collected blood samples, provided internal monitoring and additional medical data, and corrected the final manuscript. RM participated in the maintenance of the study, revising the article critically for important intellectual content, and providing final approval. All authors read and approved the manuscript.
